# A rare case of CD1a‐negative Langerhans cell histiocytosis of the central nervous system in a child

**DOI:** 10.1002/ccr3.1136

**Published:** 2017-09-01

**Authors:** Priscilla Powell, Gaile Vitug, Fernando Castro‐Silva, Anish Ray

**Affiliations:** ^1^ Weatherford Regional Medical Center Weatherford Texas; ^2^ University of Texas Health Science Center at San Antonio San Antonio Texas; ^3^ Cook Children's Medical Center Fort Worth Texas

**Keywords:** Basal ganglia, CD1a, dendritic cell disorder, diabetes insipidus, Langerhans cell histiocytosis, S100

## Abstract

Langerhans cell histiocytosis is a dendritic cell disorder with a wide spectrum of severity and presentations. Histopathology typically demonstrates a proliferation of Langerhans cells and a lymphohistiocytic inflammatory infiltrate with eosinophils. The diagnosis is supported by immunohistochemistry with the cell markers S100, CD1a, CD68, and Langerin [*Blood*, 126, 2015, 26 and *N Engl J Med*, 331, 1994, 154].

## Introduction

Langerhans cell histiocytosis (LCH) is an inflammatory and neoplastic disease. Diagnostic features include a mixed inflammatory background and a proliferation of Langerhans cells with grooved reniform nuclei expressing CD1a and CD207 (Langerin) by immunohistochemistry (IHC). The Langerhans cells demonstrate the common BRAF mutations and characteristic Birbeck granules in the cytoplasm by electron microscopy 1. This case report describes a rare pediatric case of central nervous system (CNS) LCH with CD1a‐negative Langerhans cells. To our knowledge, this is the first case of CD1a‐negative LCH in a child, confirmed by biopsy of the CNS lesion.

## Case Report

**Figure 1:**
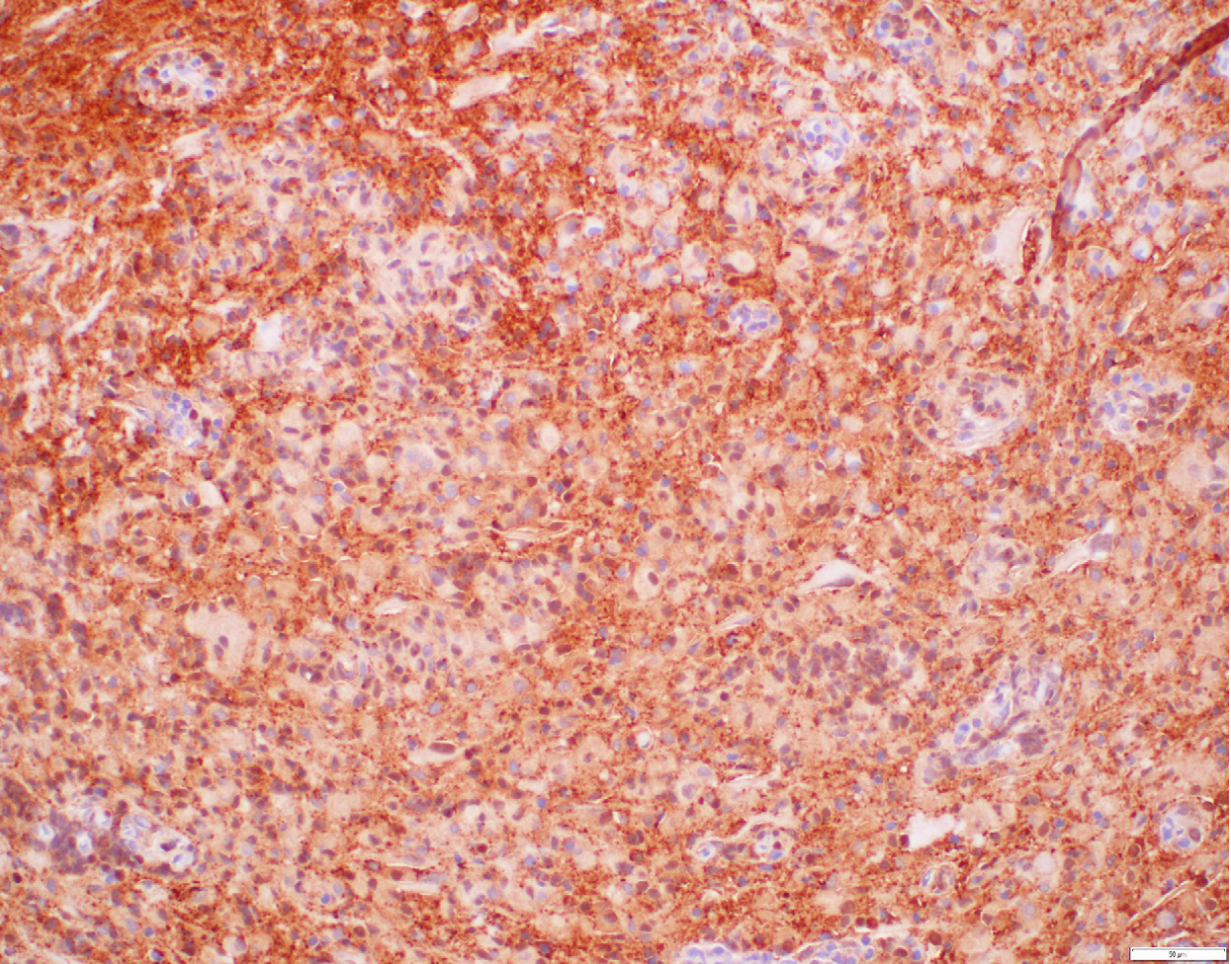
S100 immunohistochemistry. Close examination reveals that the perivascular Langerin‐positive cells express S100 protein. Note that the background glioneuronal tissue is also S100 protein positive.

**Figure 2:**
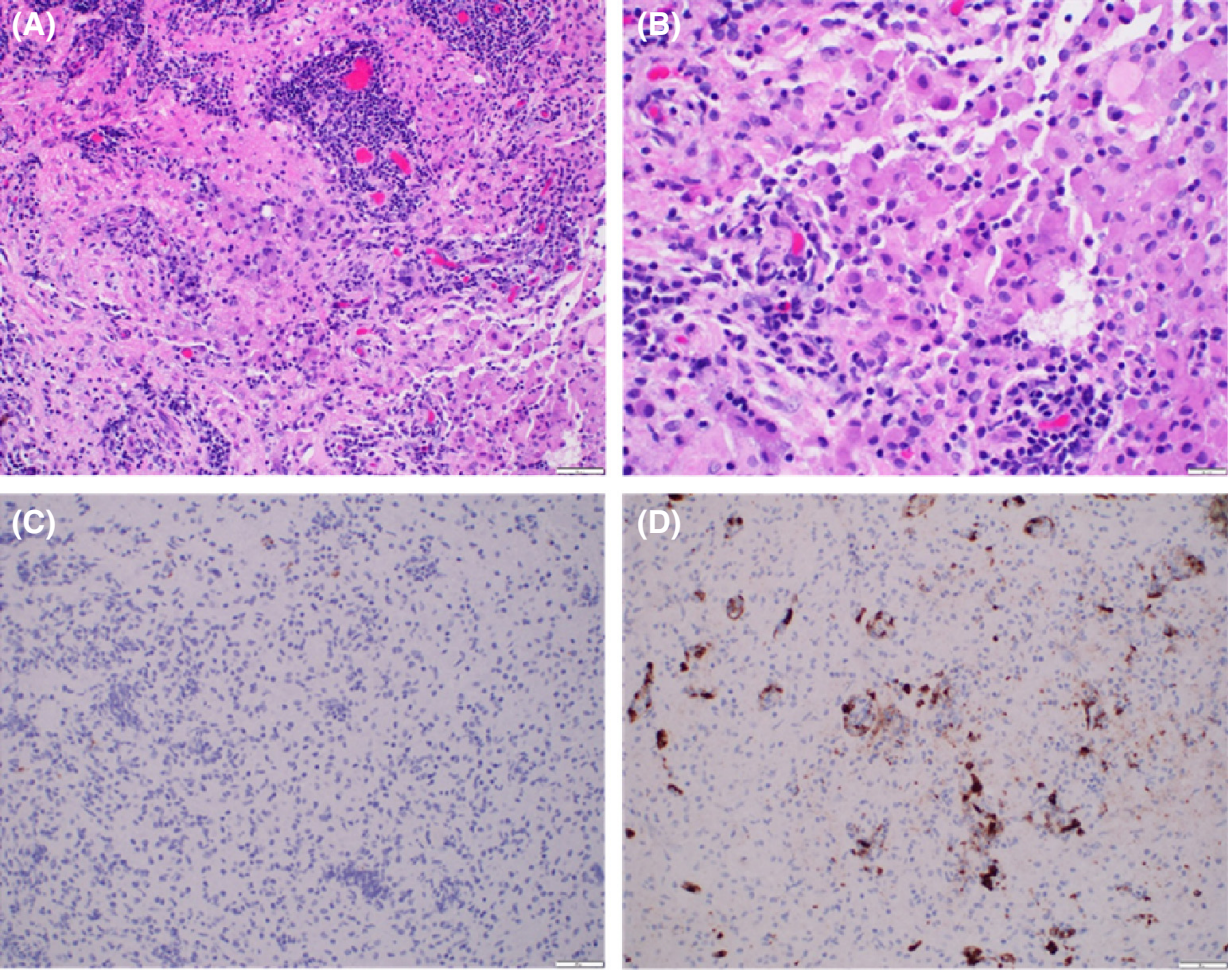
Histopathology of brain lesion. (A, B) Dendritic cell infiltration with mixed inflammation including histiocytes, lymphocytes, plasma cells, and rare eosinophils (H&E 200× and 400×). (C) Langerhans cells lack CD1a expression (200×). (D) Langerin immunohistochemistry demonstrates Langerhans cells predominantly involving the Virchow–Robin spaces (200×).

A 9‐year‐old male presented to his pediatrician with a 3‐year history of progressively worsening polyuria and polydipsia, accompanied by nocturnal enuresis. Physical examination was unremarkable. In particular, there was no evidence of ataxia, dysmetria, dysarthria, or behavior change. Blood examination demonstrated no abnormality so bone marrow biopsy was not necessary. Further workup revealed central diabetes insipidus (DI) secondary to a large brain lesion involving the right basal ganglia, with thickening of the pituitary stalk, absent posterior pituitary bright spot, and infiltration involving the cerebellum demonstrated on MRI. Differential diagnosis included intracranial infection, LCH, and germinoma. Germinoma and infection were considered less likely given the characteristic of the tumor and lack of clinical symptoms. To confound the diagnostic hypothesis, additional studies showed a lack of skeletal involvement that would be expected with LCH. A biopsy of the right basal ganglia lesion was subsequently obtained with craniotomy using the burr hole technique. Histopathologic examination showed a destructive process composed of few perivascular neoplastic Langerin‐positive, S100‐positive, CD1a‐negative Langerhans cells, and an associated brisk non‐neoplastic inflammatory infiltrate composed of predominantly macrophages (Fig. 1). Given the clinical history of DI, histopathologic findings, compatible imaging findings, and the absence of another defined process, LCH was highly favored as the most likely diagnosis. Expert second opinion (pathology) concurred with a diagnosis of LCH, and a BRAF V600E activating mutation was identified in the lesion, further supporting the diagnosis of an atypical CD1a‐negative case of LCH of the CNS (Fig. 2). His treatment course consisted of cytosine arabinoside (ara‐C) at 150 mg/m^2^ daily for 5 days, once every 4 weeks, for a total of 12 months. Supportive care consisted of pegfilgrastim to overcome bone marrow suppression and trimethoprim–sulfamethoxazole for Pneumocystis prophylaxis.

This patient's central DI continues to be well managed with desmopressin (DDAVP). Clinically, he remains stable with adequate control of DI with DDAVP and no new skeletal lesions on imaging. End‐of‐therapy imaging showed his right basal ganglia mass has decreased in dimension with substantial decrease in perifocal edema, consistent with response to chemotherapy. Planned future follow‐up consists of MRI serial imaging.

## Discussion

Langerhans cell histiocytosis has been difficult to categorize as it was first described in the nineteenth century 2, and was viewed as a clonal proliferation in the 1990s 3. Currently, it comprises a proliferation of CD1a^+^/CD207^+^ dendritic cells in a flurry of other inflammatory cells 4, 5. The pathophysiology has been further elucidated to originate from a myeloid lineage, particularly since CD207^+^, or Langerin positivity is seen in patients with high‐risk LCH in their bone marrow 4. The S100 protein, a nonspecific marker, and CD1a are the most common IHC markers used for diagnostic confirmation. CD207 (Langerin) is a useful and accepted additional confirmatory marker as it is almost exclusively expressed in LCH and only focally expressed in histiocytic sarcoma 1.

Imaging modalities for patients with suspected CNS LCH include MRI of the brain 4. Although not performed in this case, PET scans are becoming increasingly prevalent in identifying lesions not detected by other modalities and to monitor response 6. Three types of LCH CNS lesions are recognized: tumors in the cerebrum and cerebellum, mass lesions of the hypothalamic–pituitary axis (associated with DI), and neurodegenerative syndrome (NDS). The latter is associated with symptoms such as dysarthria, change in behavior, ataxia, and dysmetria, none of which were present in our patient. Regardless, treatment of NDS is particularly challenging with isolated radiographic findings in the absence of clinical symptoms 4. LCH of the CNS poses added complexity due to the fact that biopsies of active LCH lesions of the brain parenchyma are exceedingly rare and little is known about the characteristic histologic features or natural history. Due to the inherent risk in biopsying the pituitary, patients with isolated DI and thickened pituitary stalk are often treated empirically. This case is particularly unusual and important as it demonstrates the possibility of LCH without CD1a positivity in pediatric patients. One case of CD1a‐negative LCH occurred in an adult female with lesions involving the parietal and occipital bones and thickened pituitary stalk. The histopathology was unremarkable, showing a typical inflammatory infiltrate of histiocytes, lymphocytes, eosinophils, and multinucleated giant cells. However, IHC revealed histiocytes positive for CD68 and S100 but negative for CD1a 7.

Although our patient's IHC results were unusual, other features involving his clinical presentation were common in CNS LCH, such as pituitary stalk thickening combined with central DI. Associated symptoms with pituitary stalk involvement include progressive loss of the visual field and other endocrinopathies 8, 9. As other histiocytic disorders can be CD1a negative, it is prudent to rule out the non‐LCH disorders. Although there are many histiocytic disorder variants, we will focus on Erdheim–Chester disease (ECD), Juvenile Xanthogranuloma (JXG), and Rosai–Dorfman for brevity and relevancy to LCH 4, 10, 11, 12. Erdheim–Chester disease, a disease rare in pediatrics, is closely related to LCH with CD68 positivity and over half possessing the BRAF V600E mutation, but is CD1a negative 12. Also, unlike LCH, ECD will show Factor XIIIa positivity 10, 12. JXG comprises myeloid‐derived cells like LCH, but the lesional cells are thought to have a dermal macrophage origin 13. JXG cells are Factor XIII positive, CD1a negative, Langerin negative, and usually S100 negative 12. Features such as foamy histiocytes with fibrosis or xanthogranulomas help to distinguish it from LCH histologically 11. Rosai–Dorfman possesses an interesting similarity to LCH in that they are both S100 positive. However, the disorder was safely ruled out because it is Langerin and CD1a negative 12.

Mass lesions of the CNS have been treated with vinblastine/prednisone, clofarabine, cladribine, and cytarabine 14. The cytarabine‐based regimen was chosen for this patient, given its CNS penetrability, lack of clinical trials to suggest superiority of other regimens, and possible benefit against neurodegenerative LCH 4.

## Conclusion

This case highlights the complexity related to the diagnosis of LCH. LCH involving the CNS, as with our patient, carries the risk of progressive pituitary dysfunction and eventual neurodegenerative LCH 4. In the presentation of this rare finding, we describe that the diagnosis of LCH can be established even without the classic CD1a marker positivity, particularly with the advancement of molecular testing.

## Authorship

PP: wrote the first draft. AR and GV: edited. FCS: contributed pathology.

## Conflict of Interest

None declared.

## References

[1] Lau, S. K. , P. G. Chu , and L. M. Weiss . 2008 Immunohistochemical expression of Langerin in Langerhans cell histiocytosis and non‐Langerhans cell histiocytic disorders. Am. J. Surg. Pathol. 32:615–619. 18277880 10.1097/PAS.0b013e31815b212b

[2] Broadbent, V. , R. M. Egeler , and M. E. Jr Nesbit . 1994 Langerhans cell histiocytosis – clinical and epidemiological aspects. Br. J. Cancer 70:S11–S16. PMC2149699 8075001

[3] Willman, C. L. , L. Busque , B. B. Griffith , B. E. Favara , K. L. Mcclain , M. H. Duncan , et al. 1994 Langerhans’‐cell histiocytosis (Histiocytosis X) – a clonal proliferative disease. N. Engl. J. Med. 331:154–160. 8008029 10.1056/NEJM199407213310303

[4] Allen, C. E. , S. Ladisch , and K. L. Mcclain . 2015 How I treat Langerhans cell histiocytosis. Blood 126:26–35. 25827831 10.1182/blood-2014-12-569301 PMC4492195

[5] Berres, M. , M. Merad , and C.E. Allen . 2014 Progress in understanding the pathogenesis of Langerhans cell histiocytosis: back to Histiocytosis X? Br. J. Haematol. 169:3–13. 25430560 10.1111/bjh.13247 PMC5193221

[6] Phillips, M. , C. Allen , P. Gerson , and K. McClain . 2009 Comparison of FDG‐PET scans to conventional radiography and bone scans in management of Langerhans cell histiocytosis. Pediatr. Blood Cancer 52:97–101. 18951435 10.1002/pbc.21782

[7] Korti, P. , J. Erukkambattu , S. Raju , and R. Tanikella . 2015 Cd1a negative Langerhans cell histiocytosis: a case report. MRIMS J. Health Sci. 3:84–86.

[8] Ghafoori, S. , S. Mohseni , B. Larijani , and M. R. Mohaheri‐Tehrani . 2015 Pituitary stalk thickening in a case of Langerhans cell histiocytosis. Arch. Iran. Med. 18:193–195. 25773695

[9] Grois, N. G. , B. E. Favara , G. H. Mostbeck , and D. Prayer . 1998 Central nervous system disease in Langerhans cell histiocytosis. Hematol. Oncol. Clin. North Am. 12:287–305. 9561901 10.1016/s0889-8588(05)70511-6

[10] Diamond, E. L. , L. Dagna , D. M. Hyman , G. Cavalli , F. Janku , J. Estrada‐Veras , et al. 2014 Consensus guidelines for the diagnosis and clinical management of Erdheim‐Chester disease. Blood 124:483–492. 24850756 10.1182/blood-2014-03-561381 PMC4110656

[11] Haroche, J. , L. Arnaud , and Z. Amoura . 2012 Erdheim–Chester disease. Curr. Opin. Rheumatol. 24:53–59. 22089098 10.1097/BOR.0b013e32834d861d

[12] Ranganathan, S . 2016 Histiocytic proliferations. Semin. Diagn. Pathol. 33:396–409. 27720561 10.1053/j.semdp.2016.08.009

[13] Bains, A. , and D. M. Parham . 2011 Langerhans cell histiocytosis preceding the development of juvenile xanthogranuloma: a case and review of recent developments. Pediatr. Dev. Pathol. 14:480–484. 21793710 10.2350/10-12-0950-CR.1

[14] Allen, C. E. , R. Flores , R. Rauch , R. Dauser , J. C. Murray , D. Puccetti , et al. 2010 Neurodegenerative central nervous system Langerhans cell histiocytosis and coincident hydrocephalus treated with vincristine/cytosine arabinoside. Pediatr. Blood Cancer 54:416–423. 19908293 10.1002/pbc.22326 PMC3444163

